# The impact of index futures crash risk on bitcoin futures returns and volatility

**DOI:** 10.1016/j.heliyon.2024.e24126

**Published:** 2024-01-08

**Authors:** Chia-Hsien Tang, Yen-Hsien Lee, Ya-Ling Huang, You-Xuan Liu

**Affiliations:** a Economics and Management Department, Guangxi Minzu Normal University, Chongzuo, Guangxi, China; b College of Business, Department of Finance, Chung Yuan Christian University, Taiwan; c Department of Golden-Ager Industry Management, Chaoyang University of Technology, Taiwan

**Keywords:** Bitcoin futures, Index futures, Crash risk, Returns volatility, COVID-19

## Abstract

This study examines the relationship between E-mini S&P 500 futures' crash risk and Bitcoin futures' returns and volatility using data from 2017 to 2021. While E-mini S&P 500's crash risk doesn't significantly influence Bitcoin returns, it correlates with its volatility, especially during events like the COVID-19 pandemic and U.S. elections. Furthermore, as global and emerging market indices rise, Bitcoin futures volatility decreases, suggesting its role as a hedging tool. These findings are pivotal for investors aiming to construct informed trading strategies, leverage Bitcoin futures as a hedging asset during economic instability, and keep tabs on traditional market indicators like E-mini S&P 500 crash risk for anticipating fluctuations in Bitcoin futures.

## 1 Introduction

In recent years, especially with the tumultuous shifts in traditional financial markets exacerbated by crises like the COVID-19 pandemic, alternative assets, notably Bitcoin, have taken a prominent position. Launched in 2008, Bitcoin's journey from a speculative novelty to a pivotal financial instrument influencing volatility spillovers in mainstream markets has been well-documented [1,2]. The pandemic era has seen Bitcoin's ascendancy further solidified as investors pivot towards non-conventional assets to mitigate risk and enhance returns [3,4].

Although existing research illuminates the nexus between Bitcoin's spot and futures markets [5,6], a notable gap persists in understanding Bitcoin's interplay with traditional financial anchors like the E-mini S&P 500 index futures, especially during times of market upheaval. The growing global acceptance of Bitcoin, as witnessed by legislative advancements in countries like El Salvador and Germany, underscores the urgency to address this gap and understand Bitcoin's evolving role in the broader financial landscape.

Our study is set against the backdrop of the U.S., a decision rooted in its pivotal position in global finance. The U.S. not only stands as a vanguard in terms of adopting financial derivatives, including Bitcoin futures, but also crafts regulatory frameworks that have ripple effects internationally. The significance of E-mini S&P 500 index futures, inherently tied to the U.S. economic landscape, further reinforces the global relevance of our findings. Our nuanced exploration of the U.S. financial ecosystem ensures that our analysis captures the subtleties that might otherwise be overshadowed in broader, global contexts.

A salient feature of our study is the deliberate bifurcation of our analysis into two distinct phases, demarcated by the advent of 2020. The financial disruptions ushered in by the COVID-19 pandemic necessitated this approach. Our observations delineate a positive correlation between the E-mini S&P 500's crash risk and Bitcoin futures volatility before 2020, a dynamic that evolves post-2020, reflecting Bitcoin futures' altered risk-return stance.

Adding to the scholarly discourse, our research taps into a plethora of market condition indicators, encompassing Economic Policy Uncertainty (EPU), Volatility Index (VIX), liquidity metrics, and the novel Daily Infectious Disease Impact on the Stock Market Volatility Index (EMVID), to shed light on this intricate relationship under diverse market climates [[7], [8], [9], [10]].

**Fig. 1:**
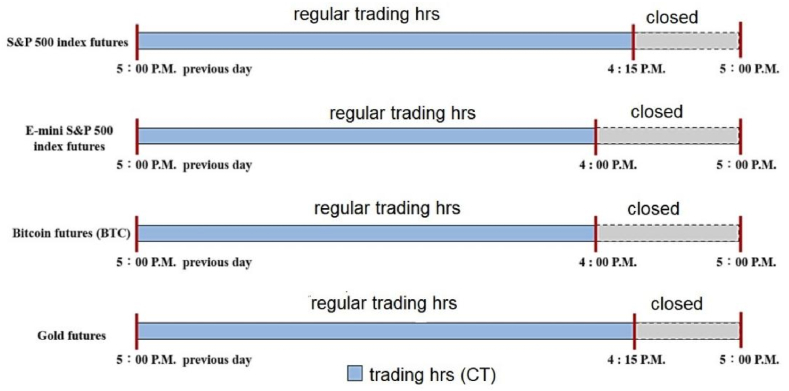
Comparison of futures market trading hours.

Our empirical investigation focused on E-mini S&P 500 and Bitcoin futures, motivated by the trading hours overlap demonstrated in Fig. 1. This figure illustrates the trading times of major futures markets, revealing a substantial concurrency in activity between these two. This temporal alignment justifies our examination of their interplay.The primary insights gleaned from our analysis reveal a nuanced impact of E-mini S&P 500 crash risk on Bitcoin futures. Specifically, while the influence on returns is limited, there exists a pronounced positive correlation with their volatility. These findings hold significant implications for investors and policymakers, providing a basis for crafting strategies adept at navigating today's financial volatility.

The rest of this paper is organized as follows: Section 2 elaborates on hypothesis development, Section 3 discusses the data and methodology employed, Section 4 presents our empirical results, and Section 5 concludes the study.

## 2 Review of relevant literature and event

### 2.1 1Bitcoin, bitcoin futures, and market crash risks

Since the advent of Bitcoin futures, their influence on price discovery has been a focal point. Using [11] information share model and Gonzalo and Granger's common factor component [12], highlighted the pivotal role of the Bitcoin futures market in price discovery [13]. further supported this by indicating a prominent information flow and price discovery from the futures to the spot market. Beyond this, Bitcoin futures also function as effective hedging tools. They bolster Bitcoin's price transparency, serving as a risk management instrument [14]. [15] further nuanced this perspective, noting the profound impacts of futures trading on the volatility dynamics of Bitcoin returns, corroborating the observations made by Ref. [16] on the effect of global price movements on the energy sector commodity during the COVID-19 pandemic and its influence on Bitcoin price movement.

Bitcoin, despite its inherent risks, has increasingly been recognized as a valuable tool for portfolio diversification [17,18]. illuminated Bitcoin's unique market position, straddling the characteristics of both gold and the U.S. dollar. This positioning suggests Bitcoin's dual utility: as a hedge against fluctuations in the U.S. dollar and a stabilizing asset for the UK stock market. Echoing this sentiment [10,19], demonstrated Bitcoin's efficacy in hedging against uncertainties in the global stock market, particularly in times of economic distress.

[20] further reinforced Bitcoin's value in diversified portfolios, showing that the inclusion of Bitcoin alongside traditional assets like gold, oil, and stocks results in a composite portfolio that is less prone to risk [21]. complemented this perspective by underscoring Bitcoin's diversification potential against various traditional currencies.

In a related vein [22], explored the intricate interplay between Bitcoin, social media, and the Covid-19 pandemic. Their findings reveal how Bitcoin gains prominence during global crises, highlighting its relevance in the face of widespread uncertainty dding a future-focused dimension [15], delved into the role of futures trading in influencing Bitcoin's volatility. Their analysis posits that the futures market is instrumental in driving Bitcoin's price dynamics, offering a critical lens through which to view Bitcoin's behavior in financial markets. These insights coalesce to depict Bitcoin not just as a speculative asset, but as a strategic hedge and diversification vehicle, responsive to both traditional market movements and global economic events.

Bitcoin futures, while advantageous as a hedging instrument, cannot be dissociated from their speculative character. Investors, in many cases, are driven by potential speculative gains when integrating Bitcoin futures into their portfolios. This speculative aspect is underscored by Ref. [23]; who elucidated the intricate relationship between trading behaviors in the futures market and Bitcoin's inherent volatility. Similarly [24], examined the volatility and return connectedness of cryptocurrency, gold, and uncertainty, focusing on the spillover effect between cryptocurrencies and other assets.

Given the above discussion on Bitcoin's hedging attributes and the speculative nature of its futures, we propose the following hypothesis.

Hypothesis 1 In times when the E-mini S&P 500 index futures show heightened volatility, indicating a potential market crash, the return rate of Bitcoin futures is likely to surge.

The advent of Bitcoin futures brought about discussions concerning their potential effects on the cryptocurrency market [25]. provided a comprehensive perspective on this subject, exploring the effect of Bitcoin futures on the cryptocurrency market from an economic efficiency lens. Short sale restrictions exist globally, affecting market dynamics and investor behavior [26]. While some researchers argue that such restrictions hinder price discovery [27], others contend they can stabilize the market by facilitating the incorporation of undisclosed negative information [28]. However, there is also a view that short sellers may contribute to excessive price declines, particularly when uninformed investors perceive these declines as signals to sell [29,30]. Consequently, the feature of short-selling in futures markets might contribute to an increased risk of market crashes.

In addition to general futures market risks, the literature has examined the correlation between financial market volatility and Bitcoin volatility [10]. found that Bitcoin gains more attention and consequently higher returns during periods of increased economic uncertainty. Yet, its speculative nature can lead to excessive volatility (Bukovina & Marticek, 2016).

[31] add a dimension to this discussion by investigating the causality and dynamic spillovers between cryptocurrencies and traditional currency markets. Their findings indicate a complex interplay where cryptocurrencies, particularly Bitcoin, are influenced by and influence traditional currency markets. This interconnection underscores the need to consider the broader market context when assessing the role of Bitcoin futures. In light of these insights, our study aims to move beyond viewing Bitcoin solely as a hedging instrument. Instead, we focus on unraveling the intricate relationship between the volatility of Bitcoin futures and traditional market instruments like E-mini S&P 500 index futures. This approach acknowledges the multifaceted role of Bitcoin in financial markets, influenced by and influencing traditional financial assets. Given these considerations, we propose a revised.

Hypothesis 2 An increase in the collapse risk of E-mini S&P 500 index futures is inversely related to Bitcoin futures volatility, indicating that rising risk in traditional markets may drive investors towards Bitcoin futures for risk diversification.

### 2.2 Uncertainty and its relationship with the futures market

The Economic Policy Uncertainty (EPU) Index, created by Baker, Bloom, and Davis in 2012, measures the impact of economic uncertainty. Investors and market analysts frequently consult this index to gauge shifts in economic conditions and the business climate. Numerous studies highlight the economic policy uncertainty Index's significant influence on financial markets, including cryptocurrencies [10,32,33].

[10] argue that uncertainty in governmental actions erodes trust in conventional currencies, making alternatives like Bitcoin more appealing. The relationship between daily economic policy uncertainty values and Bitcoin returns is positive and significant, signifying that economic instability enhances Bitcoin's allure and profitability. Contradictorily [34], found no risk spillover effects from economic policy uncertainty to Bitcoin. Such contradictory findings offer nuanced insights for investors interested in asset diversification [35]. further contend that Bitcoin serves as an effective hedge against the U.S. stock market, particularly during periods of high uncertainty.

Additionally, research indicates that economic policy uncertainty positively correlates with stock price crash risks [7,8]. Similar sentiments are echoed by Ref. [36] while examining the mutual coupling between stock market and cryptocurrencies. This positive relationship is driven by two factors: the concealment of bad s by fund managers and increased information asymmetry between insiders and external investors. Consequently, we hypothesize that heightened economic policy uncertainty levels will exacerbate the crash risk in E-mini S&P 500 futures and increase both returns and volatility in Bitcoin futures.

The Volatility Index (VIX) forecasts the market's expected volatility over a 30-day period, serving as an indicator of investor sentiment. Studies indicate that the volatility index can predict Bitcoin returns and can serve as a safe haven during market instability [19,37]. [38,39] show a positive relationship between Bitcoin returns and the volatility index, suggesting that Bitcoin offers a refuge during turbulent times.

Furthermore, research reveals a positive relationship between investor sentiment and stock market crash risks [40]; Zouaoui et al., 2011) [41]. highlighted the dependence and spillover among oil market, China's stock market, and exchange rate, hinting at the importance of understanding intertwined financial networks. Based on these findings, we posit that elevated volatility index levels will increase the crash risk in E-mini S&P 500 futures while also augmenting the returns and volatility of Bitcoin futures.

### 2.3 The impact of the COVID-19 pandemic on bitcoin

The COVID-19 pandemic led to global economic instability, resulting in market volatility and heightening financial risk. U.S. interventions, such as unlimited quantitative easing, added to this uncertainty [16,42]. emphasize the effect of global price movements on the energy sector commodity and Bitcoin price movement during the pandemi, which also plays a role in understanding the global financial dynamics during these trying times. While some studies indicate that Bitcoin has lost its hedging abilities post-pandemic [39,43], others maintain its status as a safe haven [44,45]. Drawing upon [23]; we understand the profound influence the pandemic had on volatility and trading behavior in the bitcoin futures market, reinforcing the cryptocurrency's emerging role as a hedging tool during unprecedented crises. Given these conflicting viewpoints, this study introduces the following hypothesis.

Hypothesis 3 During the COVID-19 pandemic, increased crash risk in the E-mini S&P 500 futures market will positively affect Bitcoin futures returns but decrease their volatility.

### 2.4 The impact of the US presidential election on bitcoin

Since its creation, Bitcoin has seen three U.S. presidential elections—in 2012, 2016, and 2020. Notably, each election has been accompanied by bullish trends in Bitcoin prices. The 2020 election marked the first since the advent of Bitcoin futures; thus, this study zeroes in on its impact on Bitcoin markets. Post the 2020 US elections, the cryptocurrency market exhibited noteworthy dynamics. Incorporating insights from Refs. [15,25] offers a comprehensive perspective on Bitcoin's market behavior during significant global events.

[46] explored the interactions between the U.S. stock market and various assets, including Bitcoin, oil, gold, and silver, in the aftermath of the 2016 U S. election. The study found that Donald Trump's polarizing statements caused market upheaval, leading to asset diversification and hedging. Though oil emerged as an effective short-term safe haven, its efficacy varied over time [47]. demonstrated that rising political risk indices corresponded with a significant uptick in Bitcoin returns. They proposed that Bitcoin serves as a stable hedge during politically volatile times.

Conversely, Baele et al. (2015) argued that Bitcoin's role as a hedge is limited. They stated that during periods of high market volatility and uncertainty, investors prefer safer assets over riskier options like Bitcoin. The 2020 candidate Joe Biden's proposal to hike capital gains tax rates stirred concern among crypto investors, posing a threat to market participation. As cryptocurrencies gain mainstream acceptance—supported by companies like PayPal, Square, and MicroStrategy—regulatory oversight is becoming increasingly important [48]. emphasized that regulatory changes significantly influence cryptocurrency volatility.

Hypothesis 4 During U.S. presidential elections, increased crash risk in the E-mini S&P 500 futures market is hypothesized to negatively affect Bitcoin futures returns and elevate their volatility.

## 3 Methodology

### 3.1 Data

To analyze the relationship between the crash risk of E-mini S&P 500 futures and the returns and volatility of Bitcoin futures, this research uses intraday data for both instruments. The crash risk for E-mini S&P 500 futures is determined at a 5-min frequency and subsequently transformed into a daily crash risk measure. Given the official introduction of Bitcoin futures on December 17, 2017, our data timeframe spans from this date to July 30,2021.^1^

### 3.2 Method

#### 3.2.1 Measurement of market crash risk

Our evaluation of market crash risk is grounded in established methodologies, adopting techniques from Chen et al. (2001) and Kalyvas et al. (2020). We employ two distinct methods ${\;NCSKEW}_{t}$ and ${DUVOL}_{t}$ to effectively assess market crash risk in our study.

${\text{NCSKEW}}_{t}$ focuses on capturing the negative skewness of the S&P 500 index futures' daily returns as follows:

(1) $${\text{NCSKEW}}_{t}=\frac{\;-\left[n{\left(n-1\right)}^{\frac{3}{2}}\sum r_{t}^{3}\right]}{\left[\left(n-1\right)\left(n-2\right){\left(\sum r_{t}^{2}\right)}^{\frac{3}{2}}\right]}$$

Here, n represents the number of 5-min returns within a trading day for S&P 500 index futures, and r is the return value for each of those 5-min periods. A higher ${\text{NCSKEW}}_{t}$ value implies a greater likelihood of a market crash.

${\text{DUVOL}}_{t}$, calculates the "Down-to-Up Volatility," and is used as it is less affected by extreme returns and is defined as:

(2) $${\text{DUVOL}}_{t}=\log\;\left[\frac{\left(n_{u}-1\right)\sum_{\text{DOWN}}r_{t}^{2}}{\left(n_{d}-1\right)\sum_{\text{UP}}r_{t}^{2}}\right]$$

Among that, $n_{u}$ and $n_{d}$ measures the collapse risk by taking the logarithm of the ratio of the standard deviation of down days to up days during period t. The variables UP and DOWN represent the number of days with positive and negative returns, respectively, during period t. This method separates the number of days where the return is below the period's average (DOWN days) from the number of days where the return is above the period's average (UP days). A higher value of DUVOL indicates a higher risk of market collapse.

#### 3.2.2 Measurement of economic uncertainty

The economics policy uncertainty index, originally developed by Ref. [49]; is used as a measure of economic uncertainty. This choice is substantiated by the index's widespread use among market participants and policymakers, especially in the U.S., as it provides a reliable estimate of economic risks.

#### 3.2.3 Investor sentiment

Investor sentiment is evaluated through the volatility index, frequently referred to as the fear index. This index was chosen based on its proven ability to forecast market volatility, as shown in studies like Bekaert and Hoerova (2014) and Kambouroudis and McMillan (2015).

#### 3.2.4 Liquidity

We measure liquidity using the illiquidity ratio proposed by Amihud (2002), which effectively captures the price impact of an asset. This choice is based on the validation of its effectiveness in studies such as Goyenko et al. (2009). Therefore, in this study, we adopt Amihud's (2002) measure of liquidity, which is defined as follows:

(3) $${ILLIQ}_{t}={\frac{1}{D}}_{t}\sum_{t=1}^{D}\frac{\left|{RET}_{td}\right|}{{VOLD}_{td}}$$

Where $D_{t}$ refers to the number of days with available data within the period t. $\left|{RET}_{td}\right|$ is the absolute value of the return of E-mini S&P 500 index futures over d days during period t.， VOLD is the trading volume of E-mini S&P 500 index futures. This method's measure is the ratio of the total daily trading volume to the sum of absolute returns. The higher the value of ILLIQ ratio, the poorer the liquidity.

Measurement of the daily impact of infectious diseases on stock market volatility.

With the emergence of the novel coronavirus (COVID-19) in 2019, an unprecedented global health and economic crisis has arisen. As of July 2022, the virus has infected over 550 million people worldwide and resulted in 6.34 million deaths.^2^ Due to containment policies such as economic activity restrictions, lockdowns, and travel bans, economies around the world have gradually declined. Assessing the economic impact of the COVID-19 pandemic is crucial for decision-makers and investors [50]. constructed a spaper-based infectious disease stock market volatility index using three indicators: stock market volatility, spaper-based economic policy uncertainty, and subjective uncertainty from business expectation surveys. This study will use the EMVID to measure market uncertainty caused by infectious diseases.

#### 3.2.5 Measurement of Morgan Stanley Capital International (MSCI) index returns

The Morgan Stanley Capital International (MSCI) index, launched by the US index compiler Morgan Stanley, covers different industries, countries, and regions. Many fund managers, investors, and others around the world use this index as a reference for investment, and there are also many ETFs tracking the MSCI as a benchmark. This article uses three indicators: MXWO, MSCIEF, and MSCIAC. MXWO and MSCIEF respectively represent the global and emerging markets, and the constituent stocks of MSCIAC world index cover both developed and emerging markets, with developed countries accounting for about 80 %. Developed countries include the United States, Europe, and Japan, while emerging markets include Central and South America and Asia (excluding Japan). The difference between the two markets is that the market stability in developed countries is high, while the market risk in emerging markets is larger, often accompanied by violent fluctuations. Therefore, in addition to the global index, this article will use MSCIEF and MSCIAC to differentiate whether different types of markets have different results.

(4) $${\text{MSCI}}_{t}=\frac{{\text{MSCI}}_{t}\;-\;{\text{MSCI}}_{t-1}}{{\text{MSCI}}_{t-1}}$$

### 3.3 Model

To measure whether the crash risk of E-mini S&P 500 index futures affects the returns and volatility of Bitcoin, this paper sets up the following GARCH(1,1) model:

(5) $$Y_{t}=\alpha_{0}+\alpha_{1}{CR}_{t-1}+\sum_{i=1}^{p}{\theta_{i}Y}_{t-i}+\varepsilon_{t}\;{\;\varepsilon }_{t}∼\;N\;\left(0,\sigma_{t}\right)$$

(6) $${\sigma_{t}}^{2}=\beta_{0}+\beta_{1}\sigma_{t-1}^{2}{+{\;\beta }_{2}\varepsilon_{t-1}^{2}+{\;\beta }_{3}CR}_{t-1}$$

Among that, the dependent variable of $Y_{t}$ is the return rate of Bitcoin futures. ${\sigma_{t}}^{2}$ is the volatility of Bitcoin futures.， $\sigma_{t-1}^{2}$ is ARCH parameter.， $\varepsilon_{t-1}^{2}$ is GARCH parameter. ${CR}_{t-1}$ is the price crash risk of E-mini S&P 500 index futures index during the period of t−1 measured using NCSKEW and DUVOL to calculate the risk of prices collapse and test the null hypothesis:

$$H_{0}:\alpha_{1}\;<\;0\;H_{1}:\alpha_{1}\geq 0$$

$$H_{0}:{\;\beta }_{3}\;<\;0\;H_{1}:\beta_{3}\geq 0$$

In addition, this study incorporates market conditions and examines their effects on the relationship between E-mini S&P 500 futures' crash risk and Bitcoin futures' returns and volatility. Therefore, the following GARCH (1,1) model is established:

(7) $$Y_{t}=\alpha_{0}+\alpha_{1}{\text{CR}}_{t-1}+\alpha_{2}{\text{EPU}}_{t-1}+\alpha_{3}{\text{VIX}}_{t-1}+\alpha_{4}{\text{ILLIQ}}_{t-1}+\alpha_{5}{\text{EMVID}}_{t-1}+\sum_{i=1}^{p}{\theta_{i}Y}_{t-i}+\varepsilon_{t}\;\varepsilon_{t}∼N\left(0,\sigma_{t}\right)$$

(8) $${\sigma_{t}}^{2}=\beta_{0}+\beta_{1}\sigma_{t-1}^{2}+{\beta_{2}\varepsilon_{t-1}^{2}+\beta_{3}\text{CR}}_{t-1}+\beta_{4}{\text{EPU}}_{t-1}+\beta_{5}{\text{VIX}}_{t-1}+\beta_{6}{\text{ILLIQ}}_{t-1}+\beta_{7}{\text{EMVID}}_{t-1}$$

The dependent variable $Y_{t}$ in our analysis is the return of Bitcoin futures. ${\sigma_{t}}^{2}$ is the volatility of Bitcoin futures. Where, ${CR}_{t-1}$ is E-mini S&P 500 index futures index during the

period of measured using NCSKEW and DUVOL as measured by equations (1), (2), which utilize NCSKEW and DUVOL respectively to quantify the crash risk in futures markets. ${EPU}_{t-1}$ is economy policy uncertainly index; ${VIX}_{t-1}$ denotes as volatility index; ${ILLIQ}_{t-1}$ is the liquidity indicator constructed by Amihud as calculating from equation (3); ${EMVID}_{t-1}$ is a daily infectious disease impact on stock market volatility index and test the null hypothesis:

$$H_{0}:\alpha_{1}\;<\;0\;H_{1}:\alpha_{1}\geq 0$$

$$H_{0}:{\;\beta }_{3}\;<\;0\;H_{1}:\beta_{3}\geq 0\text{.}$$

Next, this study incorporates the returns of different market indices by calculating MSCI applying from equation (4), including the MSCI World Index (MXWO), MSCI Emerging Markets Index (MSCIEF), and MSCI All Country World Index (MSCIAC), to examine whether the collapse risk of E-mini S&P 500 futures in different market types would have different impacts on the returns and volatility of Bitcoin futures. Therefore, we establish the GARCH (1,1) model, as delineated in equations (5), (6), to examine the dynamic volatility of Bitcoin futures.

(9) $$Y_{t}=\alpha_{0}+\alpha_{1}{\text{CR}}_{t-1}+\alpha_{2}{\text{MXWO}}_{t-1}+\alpha_{3}{\text{MSCIEF}}_{t-1}+\alpha_{4}{\text{MSCIAC}}_{t-1}+\sum_{i=1}^{p}{\theta_{i}Y}_{t-i}+\varepsilon_{t}\;\varepsilon_{t}∼N\left(0,\sigma_{t}\right)$$

(10) $${\sigma_{t}}^{2}=\beta_{0}+\beta_{1}\sigma_{t-1}^{2}+{\beta_{2}\varepsilon_{t-1}^{2}+\beta_{3}\text{CR}}_{t-1}+\beta_{4}{\text{MXWO}}_{t-1}+\beta_{5}{\text{MSCIEF}}_{t-1}+\beta_{6}{\text{MSCIAC}}_{t-1}$$

The dependent variable of $Y_{t}$ is the return of bitcoin futures. ${\sigma_{t}}^{2}$ is the volatility of Bitcoin futures. Within that, ${CR}_{t-1}$ is E-mini S&P 500 index futures index during the period of measured using NCSKEW and DUVOL to calculate the risk of prices collapse. ${MXWO}_{t-1}$ is the return of global index; ${MSCIEF}_{t-1}$ is the return of emerging market index; ${MSCIAC}_{t-1}$ is the developed country index return and test the null hypothesis:

$$H_{0}:\alpha_{1}\;<\;0\;H_{1}:\alpha_{1}\leq 0$$

$$H_{0}:{\;\beta }_{3}\;<\;0\;H_{1}:\beta_{3}\geq 0\text{.}$$

Finally, this study will split the period into two parts: the outbreak of the COVID-19 pandemic and the period before and after the US presidential election. We will re-examine equations (7), (8) and investigate whether the collapse risk of E-mini S&P 500 index futures during different periods will have different impacts on the returns and volatility of Bitcoin futures.

## 4 Empirical result

### 4.1 Data analysis

**Fig. 2:**
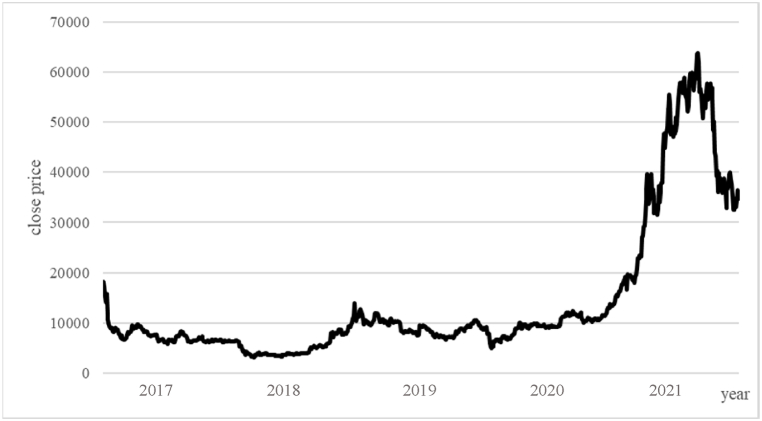
the price trend of Bitcoin futures.

**Fig. 3:**
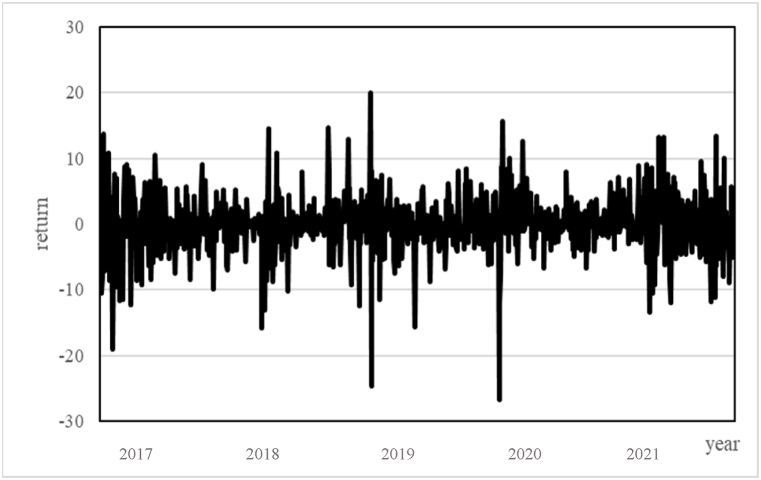
the volatility of Bitcoin futures returns.

This study's Bitcoin futures price sample covers the period from December 17, 2017, to June 30, 2021. As illustrated in Fig. 2, 'The price trend of Bitcoin futures', the analysis indicates with a peak of 63,840 points in mid-April 2021. At that time, the US was experiencing the greatest inflationary pressure in over 40 years. According to surveys, confidence ratings of leaders, including Fed Chairman Powell, President Biden, and congressional Democrats and Republicans, were below historical averages, indicating a decline in Americans' confidence in the leadership's economic management. This preliminary observation suggests that investors are likely to allocate funds to the Bitcoin futures market when faced with market economic or policy instability, which aligns with previous research findings on Bitcoin spot as a hedging or risk mitigation tool. Fig. 3 shows the volatility of Bitcoin futures returns.

### 4.2 Descriptive statistical analysis

In this section, we examine the impact of E-mini S&P 500 futures' crash risk on Bitcoin futures returns and their volatility. We use the E-mini S&P 500 index futures' intraday prices at 5-min intervals from the Tick data website to calculate crash risk. The calculation methods include DUVOL and NCSKEW, and the study period ranges from December 17, 2017, to June 30, 2021, resulting in a final sample of 884. Additionally, this study incorporates various market condition variables, including the economic policy uncertainty Index (EPU), the market volatility index (VIX), the liquidity measure (ILLIQ) constructed by Amihud (2002), the daily infectious disease impact on the stock market volatility index (EMVID), and three indices compiled by Morgan Stanley (MSCI) - MXWO, MSCIEF, and MSCIAC - to examine their potential different effects on the results.

In the analysis of the impact of E-mini S&P 500 futures' crash risk on Bitcoin futures, we refer to Table 1, which presents the basic statistics for the main variables. This includes mean, standard deviation, and other descriptive measures, providing a foundational understanding of our dataset's characteristics. The ADF test statistics in this study are all negative, with more negative values indicating a greater tendency to reject the null hypothesis.

**Table 1:** Descriptive statistical analysis This table presents the descriptive statistics of the variables used in the empirical analysis, where RET represents the daily return of bitcoin futures, DUVOL and NCSKEW are measures of E-mini S&P 500 index futures' crash risk, EPU is the index of economic policy uncertainty, VIX is the market volatility index, ILLIQ is the liquidity measure constructed by Amihud (2002), EMVID is the index measuring the impact of daily infectious diseases on stock market volatility. MXWO represents the global index return, MSCIEF represents the return of emerging market index, and MSCIAC represents the return of developed market index.

Panel A descriptive statistics							
Variable	MEAN	S.D.	MIN	MAX	Skew.	Kurt.	ADF
RET	0.107	4.885	−26.770	22.240	−0.200	7.341	−31.340
DUVOL	0.015	0.285	−1.107	0.990	−0.103	3.567	−32.740
NCSKEW	0.013	1.198	−5.564	5.428	−0.063	5.537	−31.680
EPU	156.400	121.100	10.920	807.700	1.905	6.971	−8.447
VIX	20.390	9.332	9.150	82.690	2.630	13.180	−4.077
ILLIQ	0.001	0.002	0.000	0.030	12.080	169.400	−29.970
EMVID	8.085	11.650	0.000	68.370	1.799	6.652	−7.920
MXWO	0.004	0.012	−0.099	0.088	−1.147	21.800	−33.380
MSCIEF	0.001	0.011	−0.067	0.057	−0.717	8.697	−28.160
MSCIAC	0.004	0.011	−0.095	0.084	−1.233	21.730	−32.240
Obs.	900						

### 4.3 Regression results

The results in Table 2 show that, in terms of Bitcoin futures returns, both NCSKEW and DUVOL measures are insignificant, indicating that the crash risk of E-mini S&P 500 futures has no predictive effect on Bitcoin futures returns. Past research by Ref. [51] has demonstrated that virtual currency returns are unpredictable, further supporting the empirical findings of this study. Concerning Bitcoin futures volatility, the crash risk of E-mini S&P 500 index futures exhibits a positive and significant relationship with Bitcoin futures volatility at the 1 % level. This suggests that as the crash risk of E-mini S&P 500 futures increases, the volatility of Bitcoin futures becomes larger and more intense. Previous literature has also found that due to Bitcoin's speculative nature and its increased attractiveness to investors facing market uncertainty, Bitcoin experiences excessive volatility (Bukovina and Marticek, 2016), and similar results are found in this study on the Bitcoin futures market. This finding supports Hypotheses 1 and 2, which posit a relationship between the crash risk of E-mini S&P 500 index futures (as represented by Equations (7), (8))) and the volatility of Bitcoin futures., which posit that when the crash risk of E-mini S&P 500 index futures increases, the volatility of Bitcoin futures will increase. Moreover, the ARCH coefficient estimate is significant, indicating that the previous day's Bitcoin futures return volatility affects the volatility of Bitcoin futures. The GARCH coefficient estimate is also significant, suggesting that the previous day's volatility impacts the volatility of Bitcoin futures [17], making the GARCH (1,1) model suitable for modeling Bitcoin futures volatility.

**Table 2:** The relationship between E-mini S&P 500 index futures crash risk and Bitcoin futures returns and volatility This table presents the estimation results of the impact of E-mini S&P 500 futures crash risk on Bitcoin's returns and volatility. The model is as follows: $Y_{t}$ = $\alpha_{0}$ + $\alpha_{1}{\text{CR}}_{t-1}$ + $\sum_{i=1}^{p}{\theta_{i}Y}_{t-1}$ + $\varepsilon_{t}$, ${\;\varepsilon }_{t}$ ∼ N (0, $\sigma_{t}$), and ${\sigma_{t}}^{2}$ = $\beta_{0}$ + $\beta_{1}\sigma_{t-1}^{2}{+\beta_{2}\varepsilon_{t-1}^{2}+\beta_{3}\text{CR}}_{t-1}$; $Y_{t}$ is the return of bitcoin futures, $\sigma_{t}$ is the volatility of bitcoin; ${\text{CR}}_{t-1}$ is E-mini S&P 500 index futures index during the period of measured using NCSKEW and DUVOL to calculate the risk of a prices crash. The numbers in parentheses are the p-values, where *** indicates significance at the 1 % level, ** indicates significance at the 5 % level, and * indicates significance at the 10 % level.

$Y_{t}$		
	(1)	(2)
$\alpha_{0}$	0.110	0.118
	(0.472)	(0.443)
$\alpha_{1}$	−0.164	−0.702
	(0.235)	(0.194)
$\theta_{i}$	−0.010	−0.023
	(0.807)	(0.563)
${\sigma_{t}}^{2}$		
$\beta_{0}$	0.829***	0.688***
	(0.000)	(0.002)
$\beta_{1}$	0.067***	0.075***
	(0.000)	(0.000)
$\beta_{2}$	0.894***	0.850***
	(0.000)	(0.000)
$\beta_{3}$	0.339***	1.770***
	(0.000)	(0.000)
Obs.	899	899

Table 3 and Table 4 present the main regression analysis results for equations (7), (8), examining the impact of different market conditions on the relationship between lagged crash risk of E-mini S&P 500 index futures and Bitcoin futures returns and volatility. From Table 1's correlation matrix, some variables exhibit high correlation. To avoid affecting the research results, this study uses the Variance Inflation Factor (VIF) to detect multicollinearity issues, with a VIF greater than 10 as the criterion for determining the presence of multicollinearity. After testing, only the global index returns (MXWO), emerging markets index returns (MSCIEF), and developed markets index returns (MSCIAC) exhibit excessive VIFs, while the remaining variables are below 3.57. Therefore, tables 3 and 4 only include market conditions such as economic policy uncertainty (EPU), market volatility index (VIX), liquidity indicator (ILLIQ), and daily contagion impact on stock market volatility index (EMVID).

**Table 3:** Relationship between E-mini S&P 500 Index Futures Crash Risk (measured by NCSKEW) and Bitcoin Futures Returns and Volatility with Market Condition This table presents the estimation results of the relationship between E-mini S&P 500 futures crash risk and Bitcoin futures returns and volatility, incorporating market conditions. The model is specified as follows: $Y_{t}$ = $\alpha_{0}+\alpha_{1}{CR}_{t-1}+\alpha_{2}{EPU}_{t-1}+\alpha_{3}{VIX}_{t-1}+\alpha_{4}{ILLIQ}_{t-1}+\alpha_{5}{EMVID}_{t-1}+\sum_{i=1}^{p}{\theta_{i}Y}_{t-i}+\varepsilon_{t},\varepsilon_{t}$ ∼ N (0, $\sigma_{t}$), and ${\sigma_{t}}^{2}$ = $\beta_{0}+\beta_{1}\sigma_{t-1}^{2}+{\beta_{2}\varepsilon_{t-1}^{2}+\beta_{3}CR}_{t-1}+\beta_{4}{EPU}_{t-1}+\beta_{5}{VIX}_{t-1}+\beta_{6}{ILLIQ}_{t-1}+\beta_{7}{EMVID}_{t-1};Y_{t}$ is the return of bitcoin future; ${\sigma_{t}}^{2}$ is the volatility of bitcoin. Among that, E-mini S&P 500 is the future index during t−1 by using NCSKEW to calculate the risk of a price crash. ${\text{EPU}}_{t-1}$ is the economy policy uncertainty index; ${VIX}_{t-1}$ represents as the volatility index; ${ILLIQ}_{t-1}$ is the liquidity indicator constructed by Amihud; ${EMVID}_{t-1}$ is a daily infectious disease impact on stock market volatility index. The values in parentheses are p-values, where *** indicates significance at the 1 % level, ** indicates significance at the 5 % level, and * indicates significance at the 10 % level.

Y					
	(1)	(2)	(3)	(4)	(5)
$\alpha_{0}$	−0.337	−0.393	0.107	−0.027	−0.380
	[0.169]	[0.330]	[0.508]	[0.887]	[0.445]
$\alpha_{1}$	−0.167	−0.197	−0.152	−0.172	−0.142
	[0.238]	[0.141]	[0.286]	[0.210]	[0.325]
$\alpha_{2}$	0.003**				0.004**
	[0.010]				[0.027]
$\alpha_{3}$		0.025			0.002
		[0.191]			[0.953]
$\alpha_{4}$			−0.023		−0.720
			[0.980]		[0.467]
$\alpha_{5}$				0.018	−0.015
				[0.229]	[0.557]
$\theta_{i}$	−0.035	−0.027	−0.029	−0.028	−0.042
	[0.389]	[0.512]	[0.489]	[0.499]	[0.303]
${\sigma_{t}}^{2}$					
$\beta_{0}$	0.861***	0.767***	1.080***	0.875***	1.404***
	[0.000]	[0.001]	[0.000]	[0.000]	[0.000]
$\beta_{1}$	0.827***	0.803***	0.753***	0.794***	0.738***
	[0.000]	[0.000]	[0.000]	[0.000]	[0.000]
$\beta_{2}$	0.081***	0.091***	0.097***	0.090***	0.080***
	[0.000]	[0.000]	[0.000]	[0.000]	[0.000]
$\beta_{3}$	0.320***	0.337***	0.363***	0.347***	0.415***
	[0.000]	[0.000]	[0.000]	[0.000]	[0.000]
$\beta_{4}$	−0.001**				−0.003***
	[0.016]				[0.000]
$\beta_{5}$		0.001			−0.003
		[0.807]			[0.801]
$\beta_{6}$			1.042***		1.182***
			[0.000]		[0.000]
$\beta_{7}$				0.006	0.037***
				[0.156]	[0.000]
Obs.	899	899	899	899	899

**Table 4:** After incorporating market conditions, the relationship between the crash risk of E-mini S&P 500 index futures (measured by DUVOL) and Bitcoin futures returns and volatility This table incorporates market conditions and re-examines the impact of the crash risk of E-mini S&P 500 index futures on Bitcoin returns and volatility. The model is as follows: $Y_{t}$ = $\alpha_{0}+\alpha_{1}{CR}_{t-1}+\alpha_{2}{EPU}_{t-1}+\alpha_{3}{VIX}_{t-1}+\alpha_{4}{ILLIQ}_{t-1}+\alpha_{5}{EMVID}_{t-1}+\sum_{i=1}^{p}{\theta_{i}Y}_{t-i}+\varepsilon_{t},\varepsilon_{t}$ ∼ N (0, $\sigma_{t}$), and ${\sigma_{t}}^{2}$ = $\beta_{0}+\beta_{1}\sigma_{t-1}^{2}+{\beta_{2}\varepsilon_{t-1}^{2}+\beta_{3}CR}_{t-1}+\beta_{4}{EPU}_{t-1}+\beta_{5}{VIX}_{t-1}+\beta_{6}{ILLIQ}_{t-1}+\beta_{7}{EMVID}_{t-1};{\;Y}_{t}$ is the return of bitcoin future; ${\sigma_{t}}^{2}$ is the volatility of bitcoin. Among that, ${CR}_{t-1}$ is E-mini S&P 500 is the future index during t−1 by using DUVOL to calculate the risk of a price crash. ${EPU}_{t-1}$ is the economy policy uncertainty index; ${VIX}_{t-1}$ represents as the volatility index; ${ILLIQ}_{t-1}$ is the liquidity indicator constructed by Amihud; ${EMVID}_{t-1}$ is a daily infectious disease impact on stock market volatility index. The values in parentheses are p-values, where *** indicates significance at the 1 % level, ** indicates significance at the 5 % level, and * indicates significance at the 10 % level.

$Y_{t}$					
	(1)	(2)	(3)	(4)	(5)
$\alpha_{0}$	−0.332	−0.386	0.096	−0.021	−0.360
	[0.180]	[0.335]	[0.558]	[0.914]	[0.471]
$\alpha_{1}$	−0.729	−0.864*	−0.630	−0.757	−0.638
	[0.196]	[0.098]	[0.256]	[0.162]	[0.268]
$\alpha_{2}$	0.003***				0.004**
	[0.009]				[0.028]
$\alpha_{3}$		0.025			−0.000
		[0.179]			[0.993]
$\alpha_{4}$			0.287		−0.392
			[0.751]		[0.673]
$\alpha_{5}$				0.018	−0.014
				[0.212]	[0.586]
$\theta_{1}$	−0.035	−0.027	−0.027	−0.028	−0.040
	[0.373]	[0.500]	[0.501]	[0.495]	[0.323]
${\sigma_{t}}^{2}$					
$\beta_{0}$	0.677***	0.711***	0.881***	0.729***	1.192***
	[0.002]	[0.004]	[0.000]	[0.001]	[0.000]
$\beta_{1}$	0.847***	0.818***	0.778***	0.812***	0.772***
	[0.000]	[0.000]	[0.000]	[0.000]	[0.000]
$\beta_{2}$	0.073***	0.085***	0.090***	0.084***	0.076***
	[0.000]	[0.000]	[0.000]	[0.000]	[0.000]
$\beta_{3}$	1.860***	1.652***	1.905***	1.748***	1.909***
	[0.000]	[0.000]	[0.000]	[0.000]	[0.000]
$\beta_{4}$	−0.001***				−0.003***
	[0.002]				[0.000]
$\beta_{5}$		−0.002			−0.001
		[0.762]			[0.936]
$\beta_{6}$			1.051***		1.156***
			[0.000]		[0.000]
$\beta_{7}$				0.004	0.035***
				[0.378]	[0.001]
Obs.	899	899	899	899	899

Table 3 employs the NCSKEW measure, as outlined in Equation (1), to assess the crash risk of E-mini S&P 500 index futures, incorporating different market conditions, yielding similar results to Table 2, with no significant relationship between crash risk and Bitcoin futures returns. However, economic policy uncertainty exhibits a positive and significant relationship with Bitcoin futures returns, indicating that Bitcoin futures may not be a good hedging choice under normal circumstances, but can serve as a hedging or risk management tool for investors during times of economic uncertainty. Past literature has investigated the impact of economic uncertainty on Bitcoin returns [52] and found a positive and significant relationship between economic uncertainty and Bitcoin returns. Since Bitcoin futures contracts are settled in US dollars based on the Chicago Mercantile Exchange's Bitcoin Reference Rate (BRR), Bitcoin futures results should align with past Bitcoin spot research. This study's results, similar to past literature, suggest that investors may also diversify economic uncertainty risks by investing in Bitcoin futures.

In terms of Bitcoin futures volatility, there is a positive and significant correlation with the E-mini S&P 500 index futures crash risk, consistent with Table 2's results before adding market conditions. This indicates that when the E-mini S&P 500 futures crash risk is higher, Bitcoin futures' volatility is larger and more intense. EPU is significantly negatively correlated with Bitcoin futures volatility [35]. suggest that due to increased economic uncertainty leading to increased financial asset volatility, investors seek assets with hedging and risk management functions to counteract the impact of uncertainty. Cryptocurrencies, independent of existing economic and financial systems, with Bitcoin having the highest proportion and best liquidity, can serve as hedging tools or safe havens against economic uncertainty.

Overall, when investors face uncertainties in future fiscal policies, regulations, and monetary policies, they may turn to Bitcoin as a potential hedging or risk management asset to increase returns and reduce portfolio volatility. This is consistent with this study's findings in the Bitcoin futures market. When economic uncertainty increases, Bitcoin futures' volatility becomes more stable. The impact of market volatility on Bitcoin volatility is not significant.

Regarding other market condition variables, including liquidity indicators and daily contagion effects on stock market volatility index, there is no significant impact on Bitcoin futures returns, but they exhibit varying degrees of positive and significant relationships with Bitcoin futures volatility. This suggests that when market liquidity is poor and contagion spreads, Bitcoin futures' volatility increases.

Table 4 replaces NCSKEW with DUVOL to measure E-mini S&P 500 index futures crash risk, and the results are consistent with Table 3. When E-mini S&P 500 index futures crash risk is higher, Bitcoin futures' volatility is larger and more intense. Furthermore, during periods of economic uncertainty, Bitcoin futures can provide investors with additional returns and protect against the volatility of other assets, serving as a tool for hedging, risk management, or risk mitigation.

Table 5 presents the main regression results for equations (9), (10). The three indices compiled by Morgan Stanley Capital International (MSCI) exhibit excessively high variance inflation factors (VIF) when combined with other market condition variables MSCIEF's VIF is 417.41; MXWO is 23,189.19; MSCIAC is as high as 27,565.49, indicating high multicollinearity with other variables. Therefore, these indices are tested independently. The three indices, MXWO, MSCIEF, and MSCIAC, represent global index returns, emerging market index returns, and developed market index returns, respectively. The relationship between the E-mini S&P 500 index futures crash risk and Bitcoin futures returns and volatility is examined using these three indices' returns to determine if the results are consistent across different financial markets [53]. previously studied Bitcoin's weekly returns in developed and emerging markets and found that Bitcoin could serve as a hedging asset in emerging markets, indicating different performance across various financial markets.

**Table 5:** Morgan Stanley Capital International (MSCI) Index Returns This table incorporates the index returns compiled by Morgan Stanley Capital International (MSCI) to re-examine the impact of E-mini S&P 500 index futures crash risk on Bitcoin returns and volatility. The model is as follows: $Y_{t}=\alpha_{0}+\alpha_{1}{CR}_{t-1}+\alpha_{2}{MXWO}_{t-1}+\alpha_{3}{MSCIEF}_{t-1}+\alpha_{4}{MSCIAC}_{t-1}+\sum_{i=1}^{p}{\theta_{i}Y}_{t-i}+\varepsilon_{t}$, $\varepsilon_{t}$ ∼ N (0, $\sigma_{t}$), and ${\sigma_{t}}^{2}=\beta_{0}+\beta_{1}\sigma_{t-1}^{2}+{\beta_{2}\varepsilon_{t-1}^{2}+\beta_{3}CR}_{t-1}+\beta_{4}{MXWO}_{t-1}+\beta_{5}{MSCIEF}_{t-1}+\beta_{6}{MSCIAC}_{t-1}$; $Y_{t}$ is the return of bitcoin future; ${\sigma_{t}}^{2}$ is the volatility of bitcoin. Among that, ${CR}_{t-1}$ is E-mini S&P 500 is the future index during t−1 by using NCSKEW and DUVOL to calculate the risk of a price crash. ${MXWO}_{t-1}$ is the global return index; ${MSCIEF}_{t-1}$ is the emerging market index; ${MSCIAC}_{t-1}$ is the developed country index. The values in parentheses are p-values, where *** indicates significance at the 1 % level, ** indicates significance at the 5 % level, and * indicates significance at the 10 % level. ${\sigma_{t}}^{2}$ is the volatility of bitcoin. Among that, ${CR}_{t-1}$ is E-mini S&P 500 is the future index during t−1 by using DUVOL to calculate the risk of price collapse. ${EPU}_{t-1}$ is the economy policy uncertainty index; ${VIX}_{t-1}$ represents as the volatility index; ${ILLIQ}_{t-1}$ is the liquidity indicator constructed by Amihud; ${EMVID}_{t-1}$ is a daily infectious disease impact on stock market volatility index.

$Y_{t}$						
	(1)	(2)	(3)	(4)	(5)	(6)
$\alpha_{0}$	0.098	0.099	0.098	0.106	0.105	0.105
	[0.519]	[0.499]	[0.520]	[0.493]	[0.479]	[0.495]
$\alpha_{1}$	−0.141	−0.142	−0.139	−0.622	−0.597	−0.612
	[0.372]	[0.299]	[0.365]	[0.300]	[0.267]	[0.296]
$\alpha_{2}$	4.644			3.797		
	[0.826]			[0.855]		
$\alpha_{3}$		5.652			5.768	
		[0.696]			[0.692]	
$\alpha_{4}$			5.311			4.478
			[0.801]			[0.829]
$\theta_{i}$	−0.029	−0.033	−0.030	−0.028	−0.032	−0.029
	[0.484]	[0.423]	[0.473]	[0.492]	[0.435]	[0.482]
${\sigma_{t}}^{2}$						
$\beta_{0}$	0.848***	0.783***	0.847***	0.732***	0.649***	0.730***
	[0.000]	[0.000]	[0.000]	[0.002]	[0.004]	[0.002]
$\beta_{1}$	0.803***	0.800***	0.803***	0.814***	0.813***	0.813***
	[0.000]	[0.000]	[0.000]	[0.000]	[0.000]	[0.000]
$\beta_{2}$	0.083***	0.089***	0.083***	0.079***	0.083***	0.079***
	[0.000]	[0.000]	[0.000]	[0.000]	[0.000]	[0.000]
$\beta_{3}$	0.303***	0.340***	0.305***	1.519***	1.742***	1.534***
	[0.000]	[0.000]	[0.000]	[0.001]	[0.000]	[0.001]
$\beta_{4}$	−26.589***			−26.598***		
	[0.000]			[0.000]		
$\beta_{5}$		−41.877***			−41.281***	
		[0.000]			[0.000]	
$\beta_{6}$			−28.611***			−28.632***
			[0.000]			[0.000]
Obs.	899	899	899	899	899	899

In addition, Table results also show that, regardless of whether NCSKEW or DUVOL is used to measure the E-mini S&P 500 index futures crash risk, there is no significant relationship with Bitcoin futures returns, consistent with the regression results from Table 2, Table 3, Table 4. The returns of MXWO, MSCIEF, and MSCIAC indices also have no significant relationship with Bitcoin futures. In terms of Bitcoin futures volatility, the E-mini S&P 500 index futures crash risk exhibits a positive and significant relationship with Bitcoin futures volatility, indicating that as the E-mini S&P 500 index futures crash risk increases, Bitcoin futures volatility becomes more intense. Conversely, MXWO, MSCIEF, and MSCIAC are negatively and significantly related to Bitcoin futures volatility, suggesting that as global, emerging, and developed market index returns increase, Bitcoin futures volatility decreases. This implies that during financial market volatility, Bitcoin futures can serve as a hedging or risk management tool to counteract the instability of other assets, and this effect is most pronounced in emerging markets.

### 4.4 Subsample regression results analysis

Before delving further into the revised analysis, it's pertinent to reflect on the insights provided by Fig. 1, Fig. 3, which contextualize the market trends and volatility patterns observed in our study. It's imperative to provide the rationale for selecting E-mini S&P 500 index futures and Bitcoin futures as subjects of this study. While Table 6 reveals no significant impact of the crash risk of E-mini S&P 500 index futures on Bitcoin futures returns, either pre- or post-COVID-19, the investigation holds relevance for multiple reasons.

**Table 6:** before and after the COVID-19 pandemic. This table divides the study period into before and after the COVID-19 pandemic and re-examines the impact of the crash risk of E-mini S&P 500 index futures on Bitcoin returns and volatility. The model is as follows: $Y_{t}=\alpha_{0}+\alpha_{1}{CR}_{t-1}+\alpha_{2}{EPU}_{t-1}+\alpha_{3}{VIX}_{t-1}+\alpha_{4}{ILLIQ}_{t-1}+\alpha_{5}{EMVID}_{t-1}+\sum_{i=1}^{p}{\theta_{i}Y}_{t-i}+\varepsilon_{t}$, ${\;\varepsilon }_{t}$ ∼ N (0, $\sigma_{t}$), and ${\sigma_{t}}^{2}=\beta_{0}+\beta_{1}\sigma_{t-1}^{2}+{\beta_{2}\varepsilon_{t-1}^{2}+\beta_{3}CR}_{t-1}+\beta_{4}{EPU}_{t-1}+\beta_{5}{VIX}_{t-1}+\beta_{6}{ILLIQ}_{t-1}+\beta_{7}{EMVID}_{t-1}$; $Y_{t}$ is the return of bitcoin future; ${\sigma_{t}}^{2}$ is the volatility of bitcoin. Among that, E-mini S&P 500 is the future index during t−1 by using NCSKEW to calculate the risk of a price crash. ${\text{EPU}}_{t-1}$ is the economy policy uncertainty index; ${VIX}_{t-1}$ represents as the volatility index; ${ILLIQ}_{t-1}$ is the liquidity indicator constructed by Amihud; ${EMVID}_{t-1}$ is a daily infectious disease impact on stock market volatility index. The values in parentheses are p-values, where *** indicates significance at the 1 % level, ** indicates significance at the 5 % level, and * indicates significance at the 10 % level. The values in parentheses are p-values, where *** indicates significance at the 1 % level, ** indicates significance at the 5 % level, and * indicates significance at the 10 % level.

$Y_{t}$				
	(1)	(2)	(3)	(4)
	Pre	Post	Pre	Post
$\alpha_{0}$	−0.859	0.076	−0.810	0.056
	[0.403]	[0.916]	[0.428]	[0.939]
$\alpha_{1}$	−0.088	−0.210	−0.434	−0.705
	[0.621]	[0.422]	[0.568]	[0.500]
$\alpha_{2}$	0.006	0.005***	0.005	0.005***
	[0.205]	[0.010]	[0.269]	[0.010]
$\alpha_{3}$	0.010	−0.024	0.014	−0.023
	[0.851]	[0.609]	[0.798]	[0.630]
$\alpha_{4}$	−0.676	−3.713	−0.155	−3.491
	[0.673]	[0.605]	[0.904]	[0.626]
$\alpha_{5}$	0.245	−0.000	0.089	−0.002
	[0.421]	[0.997]	[0.728]	[0.959]
$\theta_{i}$	−0.002	−0.141**	0.004	−0.140**
	[0.972]	[0.022]	[0.948]	[0.023]
${\sigma_{t}}^{2}$				
$\beta_{0}$	1.956***	2.523***	1.779***	2.486***
	[0.000]	[0.000]	[0.000]	[0.000]
$\beta_{1}$	0.501***	0.490***	0.638***	0.504***
	[0.000]	[0.000]	[0.000]	[0.000]
$\beta_{2}$	0.127***	0.043	0.101***	0.044
	[0.000]	[0.283]	[0.000]	[0.277]
$\beta_{3}$	0.341***	−0.058	2.457***	−0.420
	[0.000]	[0.653]	[0.000]	[0.382]
$\beta_{4}$	−0.002	−0.003***	−0.005*	−0.003***
	[0.324]	[0.000]	[0.066]	[0.000]
$\beta_{5}$	0.006	−0.047**	−0.001	−0.048**
	[0.747]	[0.018]	[0.943]	[0.018]
$\beta_{6}$	1.050***	9.425***	1.137***	9.127***
	[0.000]	[0.001]	[0.000]	[0.001]
$\beta_{7}$	0.246***	0.054***	0.199**	0.055***
	[0.000]	[0.000]	[0.028]	[0.000]
Obs.	514	384	514	384

Firstly, both of these financial instruments command significant attention in the global financial market, and each, in its own way, serves as a barometer for broader economic trends and investor sentiment. Secondly, the economic landscape during the COVID-19 pandemic was anything but usual. This period saw shifts in investor behavior, risk appetite, and asset allocation strategies that are worth scrutinizing, particularly for these influential financial instruments.Additionally, while our models may not have found a significant correlation between the crash risk of E-mini S&P 500 index futures and Bitcoin futures returns, they did uncover other interesting dynamics. For example, the economic policy uncertainty significantly correlates with Bitcoin returns in the post-pandemic period, hinting at Bitcoin's role as a hedging asset during times of economic instability.

Therefore, although the direct relationship between E-mini S&P 500 index futures and Bitcoin futures may not be immediately apparent, their roles as financial indicators and their dynamic behavior during the pandemic render them worthy subjects for a joint study. These markets may exhibit non-linear interactions and behaviors valuable for investors, especially in unprecedented economic conditions like the pandemic. Such findings can be critical for portfolio diversification and risk management strategies, reinforcing the significance of this research.

Previous studies, such as [47]; found different results when examining the relationship between the 2016 US presidential election and Bitcoin. They argued that political risk indices often increased due to future uncertainties, leading to a significant increase in Bitcoin returns while maintaining relatively stable volatility. This suggests that Bitcoin can be used as a hedging tool when the political situation becomes more severe. This study focuses on the impact of the 2020 US presidential election on the study results.

Table 7 divides the study period into before and after the US presidential election and re-examines equations (7), (8), using November 3, 2020, as the cut-off date when election results were confirmed. Table 7 results show that the crash risk of E-mini S&P 500 index futures has no significant effect on Bitcoin futures returns, whether before or after the US presidential election. During the election, economic policy uncertainty is positively correlated with Bitcoin futures returns, indicating that Bitcoin futures returns increase with economic policy uncertainty before the presidential candidates are determined. During the presidential election, the crash risk of E-mini S&P 500 index futures has a significant positive impact on Bitcoin futures volatility. After the election results are confirmed, the impact of E-mini S&P 500 index futures crash risk on Bitcoin futures volatility becomes insignificant. This may be related to Joe Biden's election. According to cryptocurrency media Bitcoin.com, many cryptocurrency investors expressed dissatisfaction with Joe Biden's proposal to raise the capital gains tax from 23.8 % to 43.4 %, as it would exacerbate investor concerns about taxes owed on cryptocurrencies, leading to reluctance in the cryptocurrency market. In terms of market condition variables, economic policy uncertainty continues to maintain a significant negative relationship with Bitcoin volatility, while liquidity indicators show a significant positive correlation. The daily impact of infectious diseases on stock market volatility and the volatility index are insignificant.

**Table 7:** before and after the US presidential election. This table divides the study period into before and after the US presidential election and re-examines the impact of the crash risk of E-mini S&P 500 index futures on Bitcoin returns and volatility. The model is as follows: $Y_{t}Y_{t}=\alpha_{0}+\alpha_{1}{CR}_{t-1}+\alpha_{2}{EPU}_{t-1}+\alpha_{3}{VIX}_{t-1}+\alpha_{4}{ILLIQ}_{t-1}+\alpha_{5}{EMVID}_{t-1}+\sum_{i=1}^{p}{\theta_{i}Y}_{t-i}+\varepsilon_{t}$，且 ${\;\varepsilon }_{t}$ ∼ N (0, $\sigma_{t}$) ，以及 ${\sigma_{t}}^{2}=\beta_{0}+\beta_{1}\sigma_{t-1}^{2}+{\beta_{2}\varepsilon_{t-1}^{2}+\beta_{3}CR}_{t-1}+\beta_{4}{EPU}_{t-1}+\beta_{5}{VIX}_{t-1}+\beta_{6}{ILLIQ}_{t-1}+\beta_{7}{EMVID}_{t-1}.\;Y_{t}$ is the return of bitcoin future; ${\sigma_{t}}^{2}$ is the volatility of bitcoin. Among that, E-mini S&P 500 is the future index during t−1 by using NCSKEW and DUVOL to calculate the risk of a price crash. ${\text{EPU}}_{t-1}$ is the economy policy uncertainty index; ${VIX}_{t-1}$ represents as the volatility index; ${ILLIQ}_{t-1}$ is the liquidity indicator constructed by Amihud; ${EMVID}_{t-1}$ is a daily infectious disease impact on stock market volatility index. The values in parentheses are p-values, where *** indicates significance at the 1 % level, ** indicates significance at the 5 % level, and * indicates significance at the 10 % level. The values in parentheses are p-values, where *** indicates significance at the 1 % level, ** indicates significance at the 5 % level, and * indicates significance at the 10 % level.

$Y_{t}$				
	(1)	(2)	(3)	(4)
	Pre	Post	Pre	Post
$\alpha_{0}$	−0.478	−3.596*	−0.463	−3.626*
	[0.452]	[0.066]	[0.469]	[0.061]
$\alpha_{1}$	−0.072	−0.443	−0.270	−2.392
	[0.653]	[0.177]	[0.673]	[0.138]
$\alpha_{2}$	0.005**	0.003	0.004**	0.003
	[0.032]	[0.551]	[0.046]	[0.621]
$\alpha_{3}$	0.002	0.206	0.001	0.217*
	[0.966]	[0.100]	[0.971]	[0.085]
$\alpha_{4}$	−0.872	−2.421	−0.594	−5.426
	[0.459]	[0.877]	[0.623]	[0.745]
$\alpha_{5}$	−0.032	−0.040	−0.030	−0.035
	[0.436]	[0.529]	[0.472]	[0.575]
$\theta_{i}$	−0.020	−0.190**	−0.018	−0.195**
	[0.685]	[0.026]	[0.718]	[0.022]
${\sigma_{t}}^{2}$				
$\beta_{0}$	1.304***	1.640*	1.253***	1.614**
	[0.000]	[0.061]	[0.000]	[0.048]
$\beta_{1}$	0.666***	0.518***	0.671***	0.501***
	[0.000]	[0.000]	[0.000]	[0.001]
$\beta_{2}$	0.099***	0.028	0.097***	0.041
	[0.000]	[0.668]	[0.000]	[0.566]
$\beta_{3}$	0.363***	0.206	1.700***	0.650
	[0.000]	[0.338]	[0.000]	[0.433]
$\beta_{4}$	−0.002***	−0.006*	−0.003***	−0.006*
	[0.000]	[0.066]	[0.000]	[0.081]
$\beta_{5}$	0.018	−0.013	0.020	−0.012
	[0.236]	[0.784]	[0.184]	[0.783]
$\beta_{6}$	1.173***	27.505***	1.156***	27.478***
	[0.000]	[0.000]	[0.000]	[0.000]
$\beta_{7}$	0.011	0.038	0.009	0.037
	[0.432]	[0.232]	[0.502]	[0.254]
Obs.	713	185	713	185

## 5 Conclusion and policy implications

Our study delves deep into the nuanced relationship between the crash risk of E-mini S&P 500 index futures and Bitcoin futures returns and volatility from December 17, 2017, to June 30, 2021. The evidence suggests that while the crash risk of the E-mini S&P 500 doesn't markedly influence Bitcoin returns, it does correlate positively with Bitcoin futures volatility, particularly during high-impact global events like the COVID-19 pandemic and U.S. presidential elections.

From an investment lens, this offers actionable insights. Bitcoin futures stand out as a potent hedging tool during economic turbulence, and monitoring the E-mini S&P 500 index futures' crash risk can bolster risk management strategies for those incorporating cryptocurrencies in their portfolios. This intricate interplay also underscores the pressing need for evolved financial regulations to shield investors and kindle the inception of innovative hedging tools for tackling economic uncertainties.

### 5.1 Limitations

We acknowledge certain limitations that provide avenues for future research. Relying on high-frequency intraday data precludes insights into broader macroeconomic drivers and enduring market sentiments over longer time horizons. Analyzing interactions spanning more asset classes and alternate economic settings could also reveal additional dimensions.

### 5.2 Policy implications

As cryptocurrencies gravitate towards the financial mainstream, policymakers should note their continued susceptibility to traditional market movements. Education initiatives should focus on apprising investors of these intricacies and associated risks. Regulatory emphasis must shift towards greater transparency in crypto-asset market data reporting to aid investment decisions. Most importantly, this deepening interconnectedness warrants adaptive regulations that look beyond siloed domains and take a system-wide perspective.In wrapping up, as financial markets evolve, a proactive, well-informed stance from policymakers becomes indispensable. By distilling the findings of our study and the associated risks and uncertainties, they can sculpt a more resilient, inclusive financial landscape.

Overall, our analysis highlights the need for informed, proactive policymaking as traditional and digital asset markets converge. Addressing the uncertainty and knowledge gaps identified is imperative for engendering a resilient and inclusive financial ecosystem amidst this transformation.

## Data availability statement

Data will be made available on request.

This research was funded by the National Science and Technology Council (NSTC), Taiwan, grant number NSTC-112-2410-H-033 -024 -

## CRediT authorship contribution statement

**Chia-Hsien Tang:** Writing – review & editing, Writing – original draft, Visualization, Investigation. **Yen-Hsien Lee:** Methodology, Investigation, Formal analysis. **Ya-Ling Huang:** Visualization, Validation, Supervision, Resources, Project administration. **You-Xuan Liu:** Resources, Methodology, Formal analysis, Conceptualization.

## Declaration of competing interest

The authors declare that they have no known competing financial interests or personal relationships that could have appeared to influence the work reported in this paper.

## Footnotes

1 Key data sources include:Bitcoin futures closing prices, Economic Policy Uncertainty (EPU), the Market Volatility Index (VIX), and three indices from the Morgan Stanley Capital International Index (MSCI) - sourced from Bloomberg; E-mini S&P 500 futures data and liquidity metrics, including returns and volume, procured from tickdata.com; and COVID-19 data from the U.S. Centers for Disease Control and Prevention (CDC).

2 Data source: https://www.cdc.gov.tw/.
