# Corrigendum to “The impact of index futures crash risk on bitcoin futures returns and volatility” [Heliyon Volume 10, Issue 2, January 2024, Article e24126]

**DOI:** 10.1016/j.heliyon.2025.e43495

**Published:** 2025-06-14

**Authors:** Chia-Hsien Tang, Yen-Hsien Lee, Ya-Ling Huang, You-Xuan Liu

**Affiliations:** a Economics and Management Department, Guangxi Minzu Normal University, Chongzuo, Guangxi, China; b College of Business, Department of Finance, Chung Yuan Christian University, Taiwan; c Department of Golden-Ager Industry Management, Chaoyang University of Technology, Taiwan

In the original published version of this article, the method section was missing the data source.

The original manuscript showed the Methodology as:

To analyze the relationship between the crash risk of E-mini S&P 500 futures and the returns and volatility of Bitcoin futures, this research uses intraday data for both instruments. The crash risk for E-mini S&P 500 futures is determined at a 5-min frequency and subsequently transformed into a daily crash risk measure. Given the official introduction of Bitcoin futures on December 17, 2017, our data timeframe spans from this date to July 30, 2021.^1^

The text has been updated to include the source of the data.

The corrected version of the Methodology can be found below:

To analyze the relationship between the crash risk of E-mini S&P 500 futures and the returns and volatility of Bitcoin futures, this research uses intraday data for both instruments. The crash risk for E-mini S&P 500 futures is determined at a 5-min frequency and subsequently transformed into a daily crash risk measure. Given the official introduction of Bitcoin futures on December 17, 2017, our data timeframe spans from this date to July 30, 2021. ^1^ The data was sourced from Bloomberg (https://www.bloomberg.com/) and Tick Data (https://www.tickdata.com/).

The authors apologize for the errors.

## Declaration of competing interest

The authors declare that they have no known competing financial interests or personal relationships that could have appeared to influence the work reported in this paper.

